# *LigiLactobacillus saerimneri* M-11 as a Promising Mucosal Delivery Vector for Chickens: Genomic Insights and Discriminative Modulation of Dendritic Cell Activation

**DOI:** 10.3390/vetsci12121204

**Published:** 2025-12-16

**Authors:** Sunting Ma, Haoran Qin, Shuanglin Guo, Lei Zhang, Rong Chen, Wei Ouyang, Bin Xu, Zhenzhen Zhang, Qiyan Xiong, Xing Xie, Zhixin Feng

**Affiliations:** 1 Institute of Veterinary Medicine, Jiangsu Academy of Agricultural Sciences, Key Laboratory of Veterinary Biological Engineering and Technology, Ministry of Agriculture, Nanjing 210014, China; masunting@163.com (S.M.); zhangleisxcz@163.com (L.Z.); chenronggrape@163.com (R.C.); wei_ouyang2008@163.com (W.O.); xubin9999@foxmail.com (B.X.); zzz-260@163.com (Z.Z.); qiyanxiongnj@163.com (Q.X.); 2 College of Veterinary Medicine, Yangzhou University, Yangzhou 225000, China; q17634747309@163.com; 3 College of Veterinary Medicine, Nanjing Agricultural University, Nanjing 210014, China; gsl5050@163.com

**Keywords:** *LigiLactobacillus saerimneri*, genetic safety assessment, LPXTG motif, mucosal delivery vector, dendritic cell

## Abstract

*LigiLactobacillus saerimneri* (*L. sae*) has shown considerable promise as a probiotic in recent years, particularly in poultry production. Comprehensive evaluation of its genetic functions, safety profile, and immunogenicity is essential prior to practical application. Our previous study demonstrated that the chicken-derived strain *L. sae* M-11 colonizes effectively and exhibits a favorable safety profile at adequate dosages. In this study, we further evaluated the potential of *L. sae* M-11 by analyzing its genetic basis for intestinal adaptation, metabolic features, safety risks, and suitability as a delivery vector. Comparative genomic analysis revealed that *L. sae* has evolved distinctive genetic features and functional specialization that may facilitate host adaptation. Genomic stability assessments and virulence factor screening confirmed that *L. sae* M-11 poses no substantial health risks. Furthermore, based on transmembrane protein predictions, the LPQTGE-motif protein was identified as a cell wall anchor in genetically engineered *L. sae* M-11 using immunoelectron microscopy. Notably, this delivery system selectively activated peripheral blood monocyte-derived dendritic cells (PB-MoDCs) in vitro, as evidenced by the up-regulation of maturation markers (CD83, CD80), pro-inflammatory cytokines (IL-1β, IL-6), Th1-associated IL-12, and the chemokine CXCLi1. However, it exhibited a limited antigen presentation capacity, indicated by low expression levels of CD40, MHCII, DEC205, TNF-α, and IFN-γ. The prospects and challenges associated with the application of *L. sae* M-11 have been discussed. Overall, these findings support the potential development of *L. sae* M-11 as a microbial cell factory and mucosal delivery vector.

## Simple Summary

This study focused on a less-studied *Lactobacillus* species. A strain isolated from chicken intestines in China was selected as a representative for in-depth analysis of the genetic background and potential functional properties. Using whole-genome sequencing and bioinformatics approaches, we elucidated its general genomic features. A comprehensive genetic safety assessment indicated that this strain poses no significant risk to chicken health. We identified a surface-anchored protein suitable for lactobacilli-based antigen delivery. Notably, this delivery platform can selectively activate immune cells. We also presented a balanced evaluation of the benefits and potential risks associated with this bacterium, focusing on its gut adaptation, metabolic characteristics, safety profile, and promise as an effective vaccine delivery vehicle. These findings provide a foundation for further exploration into the probiotic attributes of this *Lactobacillus* strain.

## 1. Introduction

The gut microbiota, a complex and dynamic mutualistic community, plays a pivotal role in regulating the immunometabolic homeostasis of the host [1]. Among its constituents, the family *Lactobacillaceae* within the phylum *Firmicutes* has attracted significant attention due to its diverse metabolic capabilities and host-beneficial functions [2]. Notably, certain commercially utilized lactobacilli are generally recognized as safe [3]. Recently, intestinal lactobacilli have been increasingly explored as novel candidates for delivery systems, prompting extensive research into their genetic profiles, safety, growth-promoting properties, and immunomodulatory effects.

*Ligilactobacillus*, a genus within the *Lactobacillaceae* family, exhibits a host-associated lifestyle [4]. *Ligilactobacillus saerimneri* (*L. sae*), a commensal bacterium inhabiting the gastrointestinal tract, has been isolated from diverse sources including the equine stomach [5], porcine feces [6], healthy human volunteers [7,8], chicken intestines [9,10,11,12], carnivorous zoo animals [13], and certain fermented food products [14]. Several functional attributes of *L. sae* have been documented. For instance, the strain *L. sae* 30a (ATCC 33222), with its well-characterized genome, has been shown to produce biogenic amines such as histamine, putrescine, and cadaverine [15]. The human-derived strain *L. sae* TH58 demonstrated potent tumor necrosis factor (TNF)-inhibitory activity, highlighting its potential as an immunoprobiotic candidate [7]. Furthermore, a study investigating gut microbiota in reproductive disorders reported a down-regulation of *L. sae* and other bacterial species in patients with uterine fibroids [8].

*L. sae* demonstrates significant potential in poultry health, primarily by modulating gut microbiota and metabolic functions. Multi-omics analyses reveal that its abundance positively correlates with the expression of immunomodulatory genes and material transport processes in the chicken gut [11]. Additionally, *L. sae* UO.C121 showed strong anti-*Salmonella* activity and can reshape gut bacterial structure and short-chain fatty acid (SCFA) production [10]. The chicken-derived *L. sae* M-11 exhibits robust in vitro adhesion and effective cecal colonization. Oral administration at 5 × 10^9^ CFU to one-week-old chicks inducing no adverse clinical or histopathological effects, and elicited modest non-specific immunoprotection, confirming its inherent probiotic properties [16,17].

Due to their low cost and suitability for large-scale application, lactobacilli are promising vaccine vectors in poultry production. To date, few studies have investigated the complete genome of chicken-derived *L. sae* and its immunomodulatory potential as a vaccine vector. This study expands upon prior work by elucidating the genetic background of *L. sae* M-11 and its impact on peripheral blood mononuclear cell-derived dendritic cells (PB-MoDCs). We sequenced and analyzed the complete genome of *L. sae* M-11 to assess its genetic safety and functional potential. Furthermore, an LPXTG motif, identified in transmembrane proteins, was predicted and employed for surface display in *L. sae* M-11. The immunostimulatory effects of recombinant *L. sae* strains on chicken PB-MoDCs were investigated in vitro. This study aims to provide a balanced analysis of the benefits and risks associated with *L. sae* M-11, with a focus on its gut adaptation, metabolic features, safety profile, and potential as an effective vaccine delivery vehicle.

## 2. Materials and Methods

### 2.1. Bacteria and Plasmids

*L. sae* M-11, isolated from 20-day-old chicken cecum previously, was cultured in de Man, Rogosa, and Sharpe medium (Hopebol, Qingdao, China) without shaking. The genome was extracted and sequenced by Benagen company (Wuhan, China) using Nanopore PromethION and Illumina NovaSeq 6000. Unicycler (version 0.5.0) was used to assemble the main complete and continuous contigs. The complete sequences of *L. sae* M-11 were deposited at GenBank with the accession numbers CP144759 (chromosome), CP144760, CP144761, CP144762 (three plasmids, respectively).

The inducible expression plasmid pPG612.1(1) containing xylose isomerase promoter (XYLA), USP45 signal peptide (ssUSP) and LPXTG anchor from *Lacticaseibacillus paracasei*, was purchased from MiaoLing Biology (Wuhan, China). A DNA sequence of HCE constitutive promoter, T7g10 transcriptional enhancer, ssUSP, flag tag, VP2 coding sequence of infectious bursal disease virus (a protective immunogen) and LPXTG anchor from *L.sae* M-11, were optimized based on *LigiLactobacillus* codon preference and synthesized by General Biological company (Chuzhou, China). Then the sequence was inserted into pPG612.1(1) plasmid and replaced XYLA, ssUSP and LPXTG anchor from *Lacticaseibacillus paracasei*, giving rise to pPG612-HCE-SS-VP2-LPXTG. A control plasmid, named pPG612, lacking the flag tag and VP2 coding sequence, was also constructed.

### 2.2. Ortholog Identification and Phylogenetic Tree

Genomes of lactobacilli used for unique ortholog identification were shown as Table 1. Based on all amino acid sequences of the selected species, ortholog clustering was performed using Orthofinder software (version: 23.12; parameter: - M msa). Blastp software (version: 26.0; parameter: - evalue 1 × 10^−5^- out fmt 6) was used for alignment. Software Muscle (version: 3.8.31) was used to align multiple protein sequences of single-copy orthologs. Filter the alignment results with Trimal (version: v1.4. rev22; parameter: - gt 0.2), and merge the filtered alignment results to form supergenes. A phylogenetic tree was constructed by RAxML (version: 8.2.10) software (model PROTGAMMAWAG) based on supergenes. And the other genome assembly information are as follows. *L. sae* TBRC 5746 (ASM3003565v1); *Lactobacillus crispatus* DC21.1 (ASM976920v1); *Lacticaseibacillus rhamnosus* HN067 (ASM2439741v1); *Ligilactobacillus agilis* AM_LB8 (ASM2531147v1); *Limosilactobacillus mucosae* A1 (ASM1342386v1); *Limosilactobacillus reuteri* P43 (ASM3357043v1).

### 2.3. Functional Annotation of the Genome

To obtain comprehensive functional information about the genes, several major databases were searched, including the Gene Ontology (GO) (https://geneontology.org/ (accessed on 5 May 2024)), Clusters of Orthologous Genes (COG) (https://www.ncbi.nlm.nih.gov/COG/ (accessed on 5 May 2024)), Kyoto Encyclopedia of Genes and Genomes (KEGG) and KEGG Pathway (https://www.genome.jp/kegg/ (accessed on 5 May 2024)), and the NCBI Non-Redundant Protein database (NR) (https://ftp.ncbi.nlm.nih.gov/blast/db/FASTA/ (accessed on 5 May 2024)). BLAST+ (v2.11.0+) was used to obtain gene functional annotation results.

### 2.4. Identification of Transition Elements, Antibiotic Resistance-Associated Genes, Virulence Factors, Transmembrane Protein and LPXTG

Prophages in the genome were predicted using the PhiSpy website (v4.2.21). BLAST+ (version: 2.11.0+) software and the ISfinder database were used to predict insertion sequences. Antibiotic resistance-associated genes were identified through annotation against the CARD database (http://arpcard.mcmaster.ca (accessed on 6 May 2024)). The Pathogen Host Interactions (PHI) database (http://www.phi-base.org/ (accessed on 6 May 2024)), which contains experimentally verified information, was used to identify virulence-associated genes. The Virulence Factors Database (VFDB, http://www.mgc.ac.cn/VFs/ (accessed on 6 May 2024)) was searched to identify bacterial virulence factors, along with their functions and mechanisms. TMHMM software (v2.0c) was used to identify proteins containing transmembrane helices. The CW-PRED website (http://bioinformatics.biol.uoa.gr/CW-PRED/input.jsp (accessed on 6 May 2024)), which utilizes a hidden Markov model (HMM)-based method, was used to classify cell wall-anchored proteins containing the LPXTG motif. The presence of LPXTG motifs was confirmed by PCR amplification and sequencing, performed by General Biological Company (Anhui, China).

### 2.5. Construction of Recombinant LigiLactobacillus Strains

The recombinant expression plasmids mentioned above were constructed by General Biological Company (Chuzhou, China). To construct recombinant *L. sae* M-11 strains, the plasmids were electroporated into *L. sae* M-11 as described previously [17]. The resulting recombinant strains were named p612 and p612-VP2-anchor. Bacterial pellet and supernatant samples were collected at 12 h, 18 h, and 24 h. The bacterial supernatant was treated with a TCA Protein Precipitation Kit (Sangon Biotech, Shanghai, China). The expressed protein was analyzed by Western blotting as described previously [17]. An anti-Flag tag mouse monoclonal antibody (diluted 1:3000, Beyotime Biotechnology, Shanghai, China) and IBDV VP2 polyclonal antibody (1:40) kept in our lab, were used as the primary antibodies.

### 2.6. Immuno-Electron Microscopy Analysis

The subcellular location of the fusion protein was investigated by immuno-electron microscopy using a transmission electron microscope (TEM, JEOL, Tokyo, Japan) at the Lilai Biomedicine Experiment Center (Chengdu, China). For electron staining, ultrathin sections were prepared as previously described [18] with minor modifications. Harvested cells at the logarithmic growth phase were fixed with 3% (*v*/*v*) glutaraldehyde for 10 h at 4 °C. The bacteria were washed three times with PBS. Stepwise dehydration was performed using a graded series of acetone solutions. Bacterial cells were embedded in agar resin and polymerized. The resin blocks were sectioned into ultrathin sections (60–80 nm). Sections were mounted on 300-mesh nickel grids. For etching, the grids were placed (section-side down) on a droplet of 3% (*v*/*v*) hydrogen peroxide solution on a Parafilm surface for 30 min. After being washed with water five times and then with PBS five times, sections on the grids were blocked with 1% (*v*/*v*) calf serum in PBS for 20 min at room temperature and then incubated with an anti-Flag tag mouse monoclonal antibody for 20 h at 4 °C. After washing with PBS, the sections were incubated with a colloidal gold-conjugated goat anti-mouse IgG antibody (Bioss Antibodies, Beijing, China) for 2 h at room temperature and washed again with PBS. After staining with uranyl acetate followed by lead citrate, the sections were observed using a JEM-1400FLASH TEM (JEOL Ltd., Tokyo, Japan) operated at 80 kV.

For negative staining, bacterial cells at the logarithmic growth phase were treated with 10 μg/mL lysozyme (Solarbio, Beijing, China) at 37 °C for 10 min. The collected cells were fixed with glutaraldehyde. A droplet of the fixed cell suspension was applied to a 300-mesh nickel grid. The grids were blocked with 1% (*v*/*v*) calf serum in PBS, incubated with the mouse anti-Flag tag antibody, and then with the colloidal gold-conjugated secondary antibody. After washing and fixing, the grids were negatively stained with a mixture of methylcellulose and uranyl acetate, as described previously [18], and observed using the TEM operated at 80 kV.

### 2.7. Preparation of LigiLactobacillus Strains

Recombinant *Ligilactobacillus* strains (p612-VP2-anchor and p612) were cultured in MRS broth and harvested at the mid-log phase (18 h, OD600 ≈ 4.5). Bacterial pellets were collected by centrifugation, washed three times with sterile PBS, and exposed to UV light for 20 min for inactivation. PB-MoDCs were collected and washed with fresh antibiotic-free medium. The inactivated *Ligilactobacillus* strains were added to the PB-MoDCs at a ratio of approximately 10:1 (bacteria to cells) and co-cultured for 6 h at 37 °C. After co-culture, the cells were washed three times with PBS to remove non-phagocytosed bacteria and used for subsequent phagocytosis assays, RNA extraction, and mixed lymphocyte reaction (MLR).

### 2.8. Isolation and Culture of PB-MoDCs In Vitro

Approximately 5 mL of whole blood was collected under sterile conditions from the wing veins of 4–6-week-old female black chickens (Su breed; Poultry Research Institute of the Chinese Academy of Agricultural Sciences, Yangzhou, China) using anticoagulant. The blood was diluted with an equal volume of PBS. The animal experiments were strictly conducted in compliance with animal welfare and scientific ethics principles, and were approved by the Animal Ethics Committee of Jiangsu Academy of Agricultural Sciences (Approval Number: IACUC-RE-2024-01-021). and were conducted in accordance with the guidelines of Jiangsu Province Animal Regulations (Government Decree No. 45). Peripheral blood mononuclear cells (PBMCs) were isolated by layering the diluted blood over an equal volume of Histopaque-1119 (Sigma-Aldrich, St. Louis, MO, USA), followed by centrifugation at 700× *g* for 30 min. The mononuclear cells at the interface were collected, washed, and cultured in complete RPMI-1640 medium for 6 h to allow monocyte adhesion. Non-adherent cells were then removed by gentle washing. The adherent monocytes were cultured in complete medium supplemented with 50 ng/mL recombinant chicken granulocyte-macrophage colony-stimulating factor (GM-CSF) and 50 ng/mL recombinant chicken interleukin-4 (IL-4; Kingfisher, Saint Paul, MN, USA) to differentiate them into dendritic cells. Half of the medium was replaced with fresh cytokine-containing medium on day 3. On day 6, the resulting PB-MoDCs were stimulated with lipopolysaccharide (LPS, 200 ng/mL; Sigma-Aldrich, St. Louis, MO, USA) and blank medium as positive and negative controls, respectively. CD11c, MHCII, CD40 and CD86 were analyzed via flow cytometry (BD Biosciences, San Jose, CA, USA) as previously described [19].

### 2.9. Indirect Immunofluorescence Analysis

To detect the phagocytosis of p612-VP2-anchor by PB-MoDCs, cells were seeded into 48-well plates. After co-culture with bacteria and washing, cells were fixed with 4% paraformaldehyde for 30 min at room temperature and permeabilized with 0.2% Triton X-100 for 10 min. After blocking with 0.3% bovine serum albumin (BSA) at 37 °C for 30 min, cells were incubated with a 1:300 dilution of an anti-Flag tag mouse monoclonal antibody at 37 °C for 1 h. Following washing, cells were incubated with a 1:300 dilution of an AF555-labeled donkey anti-mouse IgG (H + L) secondary antibody at 37 °C in the dark for 30 min. After final washes, cells were mounted using Antifade Mounting Medium (Beyotime Biotech, Shanghai, China) and observed under a fluorescence microscope.

### 2.10. Quantitative Real-Time PCR (qRT-PCR) for Marker Genes and Cytokine Expression in PB-MoDCs

Total RNA was extracted from chicken PB-MoDCs using a commercial kit (OMEGA BioTek, Norcross, GA, USA). Complementary DNA (cDNA) was synthesized using the PrimeScript™ RT Master Mix (TaKaRa, Dalian, China) according to the manufacturer’s instructions. The mRNA expression levels of cell marker genes, cytokines, and chemokines in PB-MoDCs were detected using qRT-PCR with ChamQ Blue Universal SYBR qPCR Master Mix (Vazyme Biotech, Nanjing, China) on a CFX96 Touch Real-Time PCR Detection System (Bio-Rad, Hercules, CA, USA). Chicken β-actin was used as the internal reference gene. The relative expression of each target gene was calculated using the 2^−ΔΔCt^ method [16]. The specific primer sequences used are listed in Online Resource S1 (Table S1). Unstimulated PB-MoDCs were used as the negative control.

### 2.11. Mixed Lymphocyte Reaction

The MLR was performed as previously described [20] with minor modifications. Briefly, stimulator cells (PB-MoDCs previously treated with *Ligilactobacillus* strains) were treated with mitomycin C and seeded in graded numbers into 96-well plates. Peripheral blood lymphocytes (PBLs), isolated from allogeneic chickens, were used as responder cells (10^5^ cells per well). Stimulator-to-responder cell ratios of 1:1, 1:10, and 1:100 were tested in a final culture volume of 200 μL. Control wells contained stimulator cells only, responder cells only, and culture medium only. After co-culture for 72 h at 37 °C, cell proliferation was assessed using a Cell Counting Kit-8 (Beyotime Biotech, Shanghai, China) according to the manufacturer’s instructions. The stimulation index (SI) was calculated using the formula: SI = (OD_sample well_ − OD_PB-MoDCs only_)/(OD_PBL only_ − OD_blank control_) [19].

### 2.12. Statistical Analysis

Experiments were repeated three times. All data were analyzed using GraphPad Prism software (version 5.0). Results are expressed as the mean ± standard deviation (SD). Statistical significance was determined by T tests for comparing two sets of measurements in the qRT-PCR experiment. And two-way ANOVA was used in the MLR experiment for its capacity to test for interactions between factors. A *p*-value of less than 0.05 (*p* < 0.05) was considered statistically significant, and a *p*-value of less than 0.01 (* *p* < 0.01) was considered highly significant. *p* < 0.001 was considered extremely significant.

## 3. Results

### 3.1. General Genomic Features of L. sae Strains

**Figure 1:**
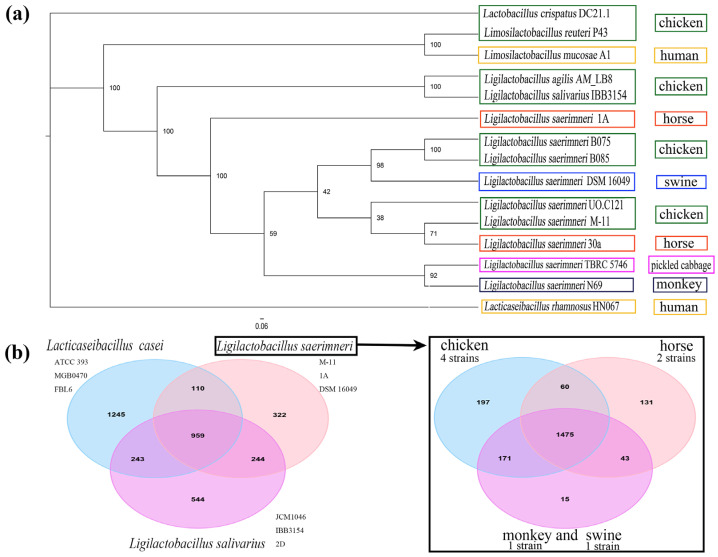
General genomic features of *L. sae* strains. (**a**) Phylogenetic tree of 15 lactobacilli based on single-copy orthologs; (**b**) Pan-genome of the *L. sae* strains. The number represents orthologous gene clusters.

To understand the genetic background of *L. sae*, all available *L. sae* genomes were analyzed comparatively. The genome sizes of *L. sae* strains are relatively small within the family *Lactobacillaceae*, and all strains contain fewer than 2000 predicted genes. The observed conservation of genome size (1.6–1.8 Mb) across strains from avian and mammalian hosts suggests a stable, niche-adapted core genome that does not appear to undergo significant expansion or contraction upon host switching. A phylogenetic tree constructed from single-copy orthologs showed that *L. sae* was closely related to *L. salivarius* (*L. sal*) and *Ligilactobacillus agilis* (Figure 1a). The clustering revealed that *L. sae* strains were not strictly segregated by host species.

A pangenome analysis was performed to assess conservation and adaptability at the species level. Figure 1b illustrated the distribution of core and dispensable gene families across the different strains. Comparative analysis showed that animal-derived *L. sae*, *L. salivarius*, and food-derived *Lacticaseibacillus casei* shared approximately 58.7% of their total gene families. Notably, *L. sae* possesses 19.7% unique gene families compared to the other two species, indicating that despite its reduced genome size, *L. sae* has evolved distinctive adaptive and functional capabilities. Furthermore, the variations in the gene families of *L. sae* strains from different host sources suggested specialized adaptations for survival in the gastrointestinal environments of both omnivores and herbivores.

To gain deeper insight into the genetic features of the chicken-derived strain *L. sae* M-11, its sequenced genome was annotated using the NCBI NR and GO databases (Online Resource S2). The NCBI NR analysis showed the majority of genes were annotated as *L. sae*, with 4.17% classified as *L. sae* 30a, a finding consistent with the phylogenetic analysis. Chicken-specific genes shared by four chicken strains were listed in Table 2. For instance, the protein WP_338434476.1 was annotated with the GO terms GO:0070007 (glutamic-type endopeptidase activity) and GO:0008233 (peptidase activity), while WP_338434480.1 was annotated with GO:0009294 (DNA-mediated transformation).

A COG analysis was performed to characterize the functional profile of proteins encoded by *L. sae* M-11. The circular genome map (Figure S1a in Online Resource S1) showed the genome-wide distribution of COG categories (Sheet S1 in Online Resource S2). As expected, most proteins were associated with core cellular functions, including “translation, ribosomal structure and biogenesis,” “amino acid transport and metabolism,” and “transcription.”

A KEGG analysis was conducted to systematically annotate the biological pathways associated with the *L. sae* M-11 proteome (Sheet S1 in Online Resource S2). Figure S1b in Online Resource S1 showed the metabolism-related pathways and the number of proteins. The top three enriched metabolic pathways were “carbohydrate metabolism,” “nucleotide metabolism,” and “amino acid metabolism.” Several proteins were also involved in “folate biosynthesis,” “vitamin B6 metabolism,” “fatty acid biosynthesis and degradation,” and “butanoate metabolism”. Beyond metabolism, proteins from *L. sae* M-11 were also linked to pathways categorized under “genetic information processing”, “environmental information processing”, “cellular processes”, and “human diseases”, as shown in Figure S1c in Online Resource S1.

### 3.2. Safety Assessment Based on L. sae M-11 Complete Genome Analysis

Genome stability, antibiotic resistance, and virulence factors are key criteria for assessing bacterial safety. Mobile genetic elements, such as plasmids, prophages, and insertion sequences (IS, a class of transposase-encoding regions), can influence genome stability. Three plasmids were predicted to encode proteins involved in replication (e.g., replication initiation protein, MobA/MobL family protein). One plasmid did not encode any essential proteins, while two others encoded hypothetical proteins that require further identification. The genome of *L. sae* M-11 contains five prophage elements; these are incomplete (each approximately 2000 bp in length) and have lost their ability to lysogenize. Six insertion sequences, designated ISLhe63, ISLac1, and IS6770, were identified within the chromosome.

To identify antibiotic resistance genes, the genome was screened against the CARD database. Only two antibiotic resistance genes were detected. A macrolide antibiotic resistance gene, belonging to the resistance-nodulation-cell division (RND) efflux pump family, was identified. Another gene conferred resistance to tetracycline antibiotics.

Virulence-associated factors were predicted using the PHI (Table 3) and VFDB (Table 4) databases. These putative factors were primarily associated with various cellular processes, including “transport and metabolism” (IMPDH, MntH1, Eno, BcaP, CvfA, PtsI, PurA, PotE, PGM, SpeC, DltA, gndA, lap, carB), “translation, ribosomal structure and biogenesis” (TufA, GidA, MnmE, RnjB, PurB), “energy production and conversion” (AtpA), “posttranslational modification, protein turnover, chaperones” (ClpX, GroEL, dnaK, clpE, clpC), “replication, recombination and repair” (CshA, Mfd), “transcription” (RpoB, relA), and “signal transduction mechanisms” (RelA, BipA, relA). Notably, no genes encoding known bacterial toxins were found.

### 3.3. Analysis of L. sae M-11 Surface Proteins and Validation of an LPXTG-Containing Protein as a Cell Wall Anchor

Transmembrane proteins have various physiological functions, including adhesion, recognition, transport, anchoring, and transduction. Software TMHMM v2.0c was used to analyze transmembrane proteins and 375 target proteins were identified (Sheet S2 in Online Resource S2). After removing the transmembrane regions of the predicted proteins mentioned above, 35 secreted proteins were found.

Cell wall-associated surface proteins displayed by Gram-positive bacteria are often implicated in communication with the environment and bacteria-host interactions. The result (Table 5) predicted by CW-PRED and TMHMM showed that three genes encoding surface proteins that belonged to the LPXTG family. All three proteins shared the identical “LPQTGE” motif.

**Figure 2:**
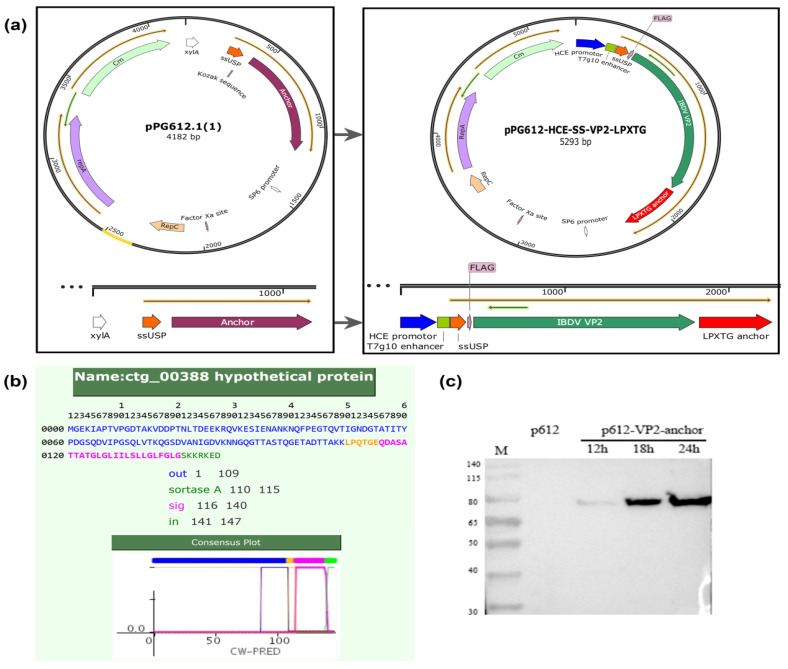
Construction of the recombinant expression plasmid containing the LPXTG motif and identification of recombinant protein expression by Western blot analysis. (**a**) Schematic diagram of the recombinant plasmid; (**b**) Prediction of LPXTG sequence by CW-PRED database. (**c**) Western blot analysis using an anti-Flag monoclonal antibody.

To explore the potential of *L. sae* M-11 for delivering exogenous proteins, we sought to confirm whether one of the predicted LPXTG-containing proteins could serve as a cell wall anchor. A recombinant expression plasmid was constructed and verified. A Flag tag and VP2 were fused to the N-terminus of this LPXTG-containing protein (Figure 2a). The transmembrane protein (ctg_00388, Figure 2b) was chosen for this study due to its single transmembrane region, which makes it a suitable candidate for testing the anchor function. The recombinant plasmids were transformed into *L. sae* M-11, and the expression of the recombinant protein was determined by Western blotting.

As shown in Figure 2c, a protein of approximately 75 kDa was detected in the bacterial pellet, which was consistent with the predicted molecular weight of the fusion protein (LPXTG anchor: ~20 kDa; Flag tag and VP2: ~55 kDa). A single immunoreactive band was observed, and its expression level showed an increasing trend over time: 24 h > 18 h > 12 h. No corresponding immunoreactive band was detected in the negative control strain harboring the empty pPG612 vector. A similar size of band was also observed in the Western blot using an anti-VP2 polyclonal antibody (Figure S2 in Online Resource S1). No immunoreactive band was detected in the bacterial supernatant. These results indicate that the protein of interest was successfully expressed and located in the bacterial pellet of *L. sae* M-11. Furthermore, the anti-Flag monoclonal antibody specifically recognized the fusion protein and did not bind to other cellular constituents.

**Figure 3:**
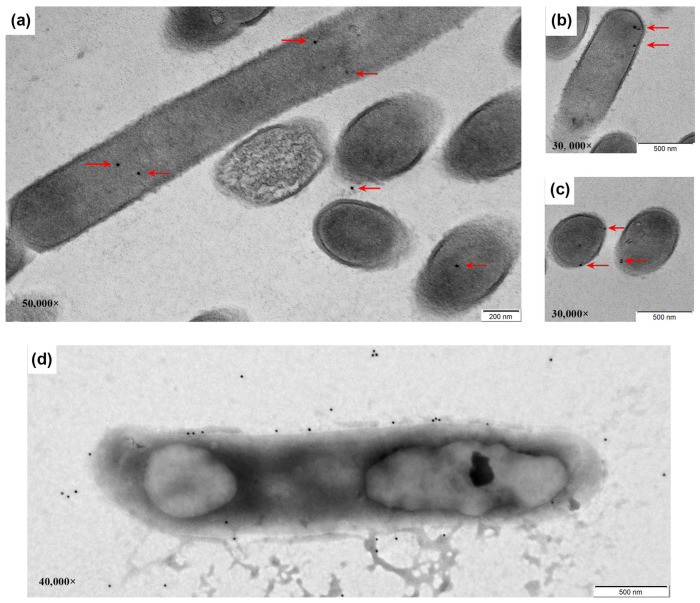
Localization of the recombinant protein by immunoelectron microscopy. (**a**–**c**) Ultrathin section with electron staining; (**d**) Negative staining of mutanolysin-treated cells. An anti-Flag monoclonal antibody was used as the primary antibody. Arrows indicate colloidal gold particles, which were bound to the recombinant protein via a goat anti-mouse secondary antibody.

To further determine the subcellular localization of the exogenous fusion protein, immunoelectron microscopy was performed using a TEM following electron and negative staining. Colloidal gold particles, which labeled the protein of interest, were observed primarily in the cytoplasm of *L. sae* M-11 (Figure 3a,b). Very few gold particles were found in the external environment (Figure 3a). Figure 3c showed that some particles were located on the cell wall. For negative staining, samples were first treated with lysozyme to induce partial cell wall rupture. Following this treatment, most gold particles were released into the external environment, while some remained associated with the inner side of the cell wall layer (Figure 3d).

Collectively, these results demonstrated that the recombinant protein was successfully expressed in *L. sae* M-11 and provided evidence that the LPXTG motif can facilitate cell wall anchoring.

### 3.4. Effects of Recombinant LigiLactobacillus Strains on PB-MoDCs

**Figure 4:**
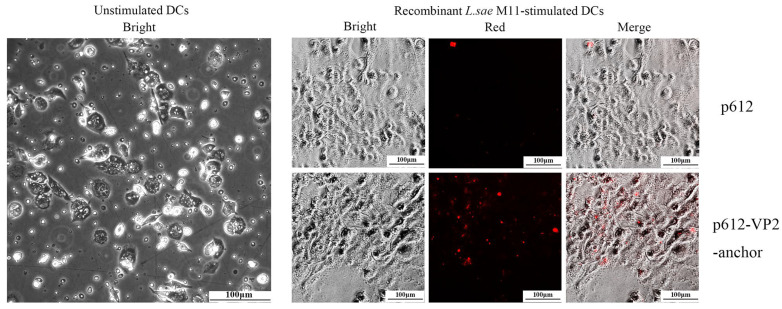
Internalization of recombinant *L. sae* M-11 by immature PB-MoDCs visualized by fluorescence microscopy. An anti-Flag monoclonal antibody was used as the primary antibody (100×).

Commensal lactobacilli help regulate and fine-tune the host’s immune system to maintain its balance. Investigating whether recombinant *L. sae* M-11 can activate PB-MoDCs is of crucial importance. Peripheral blood mononuclear cells were isolated and cultured with GM-CSF and IL-4. As shown in Figure 4, non-stimulated cells became loosely adherent by day 6 and most displayed dendritic appearance, which is characteristic of immature PB-MoDCs. LPS was used as a positive control to verify the successful maturation of the DCs. Flow cytometry analyses confirmed that LPS-stimulated DCs significantly upregulated the expression of CD11c, CD40, CD86 and MHCII. And LPS effectively induced significant proliferation in mixed lymphocyte reactions. The above results confirmed the functional maturity of the isolated PB-MoDCs (Figure S3 in Online Resource S1). Then immature PB-MoDCs were used for subsequent experiments. To further investigate whether PB-MoDCs can internalize recombinant *L. sae* M-11 after a 6-h incubation, immunofluorescence assay was performed. Figure 4 showed p612-VP2-anchor can be ingested by PB-MoDCs. As a control, the p612 group (without flag tag) showed little red fluorescence.

**Figure 5:**
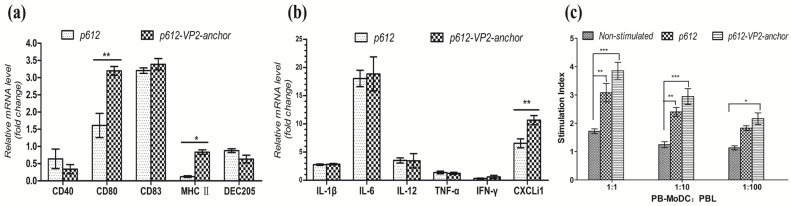
Analysis of PB-MoDC phenotype (maturation markers and cytokine mRNA) and functional capacity (mixed lymphocyte reaction) following stimulation with *L. sae*. (**a**) The mRNA levels of PB-MoDC markers following stimulation with *L. sae* assessed by relative qRT-PCR; (**b**) The mRNA levels of PB-MoDC markers following stimulation with *L. sae* assessed by qRT-PCR; (**c**) Mixed lymphocyte reaction. Bars represent the mean ± SD value of each group (* *p* < 0.05, ** *p* < 0.01, *** *p* < 0.001).

Following internalization, DCs process antigens from *L. sae*, which dictates the subsequent immune outcome: either tolerance or activation. Thus we evaluated the mRNA of DC markers and cytokines when they were stimulated by *L. sae*. The p612 group showed high CD83, CD80, pro-inflammatory cytokines (IL-1β, and IL-6), Th1-associated IL-12, chemokine (CXCLi1) mRNA levels. Conversely, low mRNA levels were observed for CD40, MHCII, DEC205, pro-inflammatory TNF-α, Th1-associated IFN-γ (Figure 5a,b). This pattern suggested that *L. sae*-treated DCs exhibited a “semi-mature” phenotype. When *L. sae* expressed IBDV VP3 as an exogenous immunogen, the mRNA levels of CD80, MHCII and the CXCLi1 were significantly higher than those in the p612 group. In the MLR assay (Figure 5c), non-stimulated PB-MoDCs weakly stimulated allogeneic cells. After *L. sae* stimulation, PB-MoDCs exhibited a significant increase in their allostimulatory capacity in the p612-VP2-anchor group (*p* < 0.001) and p612 group (*p* < 0.01), compared to the non-stimulated group, at 1:1 and 1:10 ratios of PB-MoDCs to allogeneic PBLs. At the 1:100 ratio, a significant allostimulatory capacity was observed in the p612-VP2-anchor group (*p* < 0.05). Although the mean values of the p612-VP2-anchor group were higher than those of the p612 group across all three ratios, the differences between the two groups were not statistically significant.

From the above results, it can be concluded that DCs stimulated by *L. sae* M-11 can adopt a distinctive “semi-mature” state, allowing the immune cells to identify antigens. Thus, *L. sae* can serve as a delivery vector capable of modulating DCs activation selectively.

## 4. Discussion

Lactobacilli are recognized as important and beneficial participants in the gut microbial flora, immune stimulation, and the restoration of gastrointestinal barrier function [2]. Despite recent reports highlighting the significant role of *L. sae* in the chicken gut [10,11], the current lack of comprehensive genomic data hinders its reliable application. Therefore, we conducted a genomic safety evaluation and functional annotation of *L. sae* M-11. Furthermore, we proposed an LPXTG motif-anchoring system for *L. sae* M-11 and assessed its ability to activate DCs in vitro. This approach allows us to selectively clarify the functions of chicken-derived *L. sae* and discuss its potential applications with a clear rationale.

The healthy gut is a highly competitive ecosystem. It is challenging for bacteria, even engineered probiotics, to easily occupy a niche and establish long-term colonization within a stable microbial community. This mechanism is known as “colonization resistance” [21]. To maintain a competitive advantage, intestinal lactobacilli must continually evolve. Genome decay can drive the loss of dispensable functions and promote the retention of those related to ecological fitness [22]. *L. sae* primarily exists in the gastrointestinal tract of vertebrates. Chicken-derived *L. sae* develops adaptive fine-tuning to enhance its survival in the avian gut. These genes, likely acquired via horizontal gene transfer, appear to have been favored by natural selection. For example, PrsW, a gene required for colonization, and conferring resistance to host antimicrobial peptides in Clostridium difficile [23], was identified as a chicken *L. sae*-specific gene. Its function requires further validation and could become a key factor in enhancing intestinal colonization in chickens.

The adaptive fine-tuning of lactobacilli involves the acquisition of DNA fragments from other gut bacteria, including pathogens. Therefore, we evaluated the genetic safety. Genome stability is crucial for reliable genetic transmission and cellular survival. Mobile genetic elements can assist bacteria in adaptation and be developed as tools for bacterial genome editing. However, the acquisition of antibiotic resistance genes poses potential risks. A tetracycline resistance gene, relevant to an antibiotic commonly used in Chinese poultry farming, was identified in the *L. sae* M-11 genome. We recommend using this genomic resistance locus for marker-free gene insertion in future studies, providing a stable site and facilitating phenotypic screening based on the loss of antibiotic resistance. *L. sae* M-11 was insensitive to gentamicin, neomycin, kanamycin, ampicillin, cefaclor, amoxicillin, and oxacillin [9], yet no associated resistance genes were identified. It was sensitive to penicillin and vancomycin, despite the presence of genes annotated in KEGG pathways related to “beta-Lactam resistance” and “vancomycin resistance”. The divergence between the phenotype of resistance and the absence of known genetic determinants indicates that bacteria might employ non-heritable mechanisms or poorly characterized genetic pathways to overcome antibiotics. Virulence factors typically include bacterial toxins, proteins involved in bacterial adhesion, cell surface components that offer protection, and pathogenicity-associated hydrolytic enzymes [24]. *L. sae* M-11 possessed non-classical virulence-associated factors. Based on the current evidence, *L. sae* M-11 appears to pose no significant threat to host health.

Evidence supports the significant role of lactobacilli in nutrient utilization. Our data showed *L. sae* M-11 was involved in carbohydrate, pyruvate, and amino acid metabolism. Pyruvate is further converted into various SCFAs by the intestinal microbiota. SCFAs, which are primarily responsible for energy supply, can maintain integrity of the intestinal barrier, and prevent inflammation [25]. Regarding amino acid metabolism, *L. sae* M-11 is involved in lysine biosynthesis via the diaminopimelate pathway. Lysine is one of the essential amino acids with important physiological functions, influencing chicken growth performance, intestinal health, and carcass traits [26]. In addition, *L. sae* M-11 possesses nine known glycoside hydrolases and 12 glycosyltransferases, which participate in the metabolism and utilization of dietary polysaccharides, assisting the digestive system in degrading carbohydrates.

Similarly to *L. sae* 30a, *L. sae* M-11 encodes ornithine decarboxylase, an enzyme that catalyzes the production of putrescine—a physiologically active amine involved in various cellular processes (e.g., cell proliferation) [27]. Although avian tissues possess endogenous ornithine decarboxylase activity, dietary supplementation with appropriate levels of putrescine has been shown to facilitate recovery of the intestinal epithelium post-infection and enhance meat quality and growth performance in chickens [28]. Another research showed the chicken cecal microbiota, during an inflammatory state, might maintain homeostasis by producing putrescine, bacitracin, and flavonoids [29]. However, excessive biogenic amines can be harmful to animal health and have been implicated in food poisoning incidents [30]. Therefore, the potential of *L. sae* M-11 as a feed additive, particularly its effects on growth performance and any potential toxicity, requires further investigation.

Lactobacilli possess a versatile capacity for nutrient metabolism, which has garnered attention for their development as cell factories for industrial enzymes. For instance, *L. sae* TBRC 5746 produced high titers of D-lactic acid, demonstrating its potential for industrial production [31]. The feasibility of displaying active recombinant enzymes on the surface of lactobacilli has been extensively studied. One example is the display of α-amylase on the surface of *Lacticaseibacillus casei* using an N-terminal transmembrane PgsA anchor. The recombinant strain successfully converted soluble starch to lactic acid [32]. Although lactobacilli may not be the most efficient secretors, their innate ability to anchor and/or secrete various enzymes simultaneously provides significant opportunities for such applications [33].

Moreover, lactobacilli have shown great promise as mucosal vaccine vectors over the past two decades. The specific bacterial strain and the subcellular location of antigen expression (secreted, intracellular, or surface-displayed) can significantly influence the type of immune response induced [34]. For certain applications, surface display is the preferred strategy, as the cell wall components of lactobacilli can act as natural adjuvants. For example, delivery of the human papillomavirus E7 antigen via surface display elicits a stronger immune response compared to secretion or cytosolic expression [35]. Although secreted proteins are more accessible to host antigen-presenting cells, they are often more prone to degradation than surface-anchored proteins.

Among the various cell surface anchoring systems in lactobacilli, proteins with the LPXTG motif are anchored more externally on the cell wall compared to those fused to N-terminal transmembrane or lipoprotein anchors [33]. LPXTG motif proteins contain a C-terminal cell wall sorting signal (LPXTG motif, a hydrophobic domain, and a positively charged tail). These proteins are recognized by sortase A, which catalyzes their covalent attachment to peptidoglycan [36]. In addition to the localization and stability of the expressed protein, the expression level is also crucial. Therefore, when designing lactobacilli-based delivery strategies, multiple factors must be considered. Particular attention should be given to whether the bacterial strain itself acts primarily as an immune activator or an immune regulator.

Under healthy conditions, intestinal DCs can extend their dendrites directly into the intestinal lumen, where they capture live commensal bacteria without triggering an inflammatory response. These bacteria-bearing DCs can initiate the production of protective secretory IgA (sIgA) and promote immune tolerance. sIgA neutralizes commensal bacteria and prevents their contact with epithelial cells. This process represents a form of non-inflammatory immune surveillance [37]. Once the population of commensal microbiota increase abnormally, homeostatic mechanisms can be breached and immune activation can be triggered.

Ideally, when gut probiotics are employed as delivery vehicles for immunogens, they can efficiently adapt to the intestinal environment and establish robust colonization compared with transient probiotics. This enhances their engagement with DCs, ultimately eliciting antigen-specific immune responses. Our study suggested DCs stimulated by a certain dosage of *L. sae*, can adopt a distinctive “semi-mature” state. The expression of pro-inflammatory cytokines, Th1-associated cytokines, chemokines and surface markers related to DCs maturation were selectively up-regulated. This specialized activation state enables DCs to effectively recognize *L. sae* expressing the exogenous immunogen. And it is functionally distinct from the classical semi-mature DCs, which are defined by intermediate or low levels of costimulatory molecule expression and immunological tolerance induction. The upregulation of CD80 directly provides the essential costimulatory signal (second signal) for T cell activation, while the upregulation of CD83 strongly indicates that DCs are in a functionally active state. However, the antigen presentation capability of *L. sae*-stimulated DCs appears suboptimal, as indicated by low expression levels of key molecules like CD40 and MHCII. Furthermore, the low level of Th1-associated IFN-γ may lead to inadequate clonal expansion of immunogen-specific effector cells. Hence, co-administration with suitable adjuvants or DC-targeting ligands is recommended to enhance immunogenicity.

Despite possessing the core characteristics of DCs and the advantage of convenient sourcing, PB-MoDCs may exhibit phenotypic and functional differences compared to specific intestinal DC subsets. Consequently, the present study is positioned as an initial exploration of the interplay between intestinal *L. sae* M-11 and the immune system. Future studies will involve more comprehensive assessments, evaluating the efficacy of the bacteria in combination with various adjuvants or ligands in both in vitro and in vivo models. Close attention should also be paid to how this strain interacts with other commensals and whether it can be used in combination with other probiotics to support microbial diversity while achieving targeted immune outcomes.

## 5. Conclusions

Based on the genetic characteristics, we demonstrated that *L. sae* M-11 was highly adapted to the chicken gut and functioned as a key participant in multiple metabolic pathways, particularly those involved in nutrient utilization and conversion. Although *L. sae* M-11 posed no evident health risks, the production of putrescine should be evaluated and antibiotic resistance gene requires future replacement with a marker-free system. Furthermore, the LPQTGE-motif protein in *L. sae* M-11 was characterized as a cell wall anchor and employed in our delivery system. This system selectively activated PB-MoDCs. However, it led to reduced antigen presentation capability. In conclusion, *Ligilactobacillus saerimneri* M-11 demonstrates genetic stability, host adaptation, and safety suitable for development as a mucosal delivery platform. The LPQTGE-anchored display system enables selective dendritic cell activation, although further optimization is needed to enhance antigen presentation and immunogenicity.

## Figures and Tables

**Table 1:** Genomes of lactobacilli used for unique ortholog identification.

Stain	Isolation Source	Origin	Genome Size (Mb)	Level	GenBank Accession
*L. sae*					
M-11	chicken cecum	China	1.6	Complete	GCA_036689625.1
UO.C121	digestive mucosa of broilers	Canada	1.7	Contig	GCA_026890865.1
B085	ileum of laying hens		1.7	Contig	GCA_947381505.1
B075	crop of laying hens		1.6	Contig	GCA_947381525.1
1A	*Equus caballus*	USA	1.8	Complete	GCA_013487945.1
30a	horse stomach	Australia	1.6	Contig	GCA_000317165.1
DSM 16049	pig feces	Sweden	1.7	Contig	GCA_001435165.1
N69	*Macaca fascicularis* feces	China	1.7	Contig	GCA_018917225.1
*Ligilactobacillus salivarius* (*L. sal*)					
JCM1046	swine intestine	Japan	2.3	Complete	GCA_000758365.1
IBB3154	hen	Poland	2.2	Complete	GCA_011045395.1
2D	*Equus caballus*	USA	2.0	Complete	GCA_013487885.1
*Lacticaseibacillus casei* (*L. casei*)					
ATCC393	cheese		3.0	Complete	GCA_000829055.1
MGB0470	kimchi	South Korea	2.9	Complete	GCA_015476095.1
FBL6	raw milk	South Korea	3.1	Complete	GCA_018363095.1

**Table 2:** List of chicken-specific genes in *L. sae* M-11 genome.

Ortholog No.	Locus (in M-11)	Product (in M-11)
OG0001162	WP_338434564.1	hypothetical protein
OG0001162	WP_338433801.1	helix-turn-helix domain-containing protein
OG0001356	WP_338434474.1	zinc-ribbon domain-containing protein
OG0001357	WP_338434475.1	zinc-ribbon domain-containing protein
OG0001358	WP_338434476.1	PrsW family glutamic-type intramembrane protease
OG0001359	WP_338434478.1	hypothetical protein
OG0001360	WP_338434480.1	DNA-processing protein DprA

**Table 3:** Virulence-associated genes analyzed by Pathogen Host Interactions Database.

SeqID	Gene	Gene Function
ctg_00141	*IMPDH*	Inosine-5′-monophosphate dehydrogenase
ctg_00883	*TufA*	Elongation factor Tu
ctg_00706	*MntH1*	Divalent metal cation transporter
ctg_01023	*AtpA*	ATP synthase subunit alpha
ctg_01419	*GidA*	tRNA uridine 5-carboxymethylaminomethyl modification enzyme
ctg_00380	*Eno*	Enolase
ctg_00509	*BcaP*	BCAA transporter
ctg_01057	*CvfA*	RNA modification enzyme regulating RNA degradation, Ribonuclease Y
ctg_01106	*PtsI*	Phosphoenolpyruvate-protein phosphotransferase
ctg_01420	*MnmE*	Central tRNA-modifying GTPase
ctg_00881	*ClpX*	Part of proteolytic complex
ctg_00255	*CshA*	RNA Helicase
ctg_00046	*PurA*	Adenylosuccinate synthetase
ctg_00915	*RnjB*	RNase J2
ctg_00516	*PurB*	Adenylosuccinate lyase
ctg_00353	*GroEL*	folding of newly synthesized proteins, preventing misfolding and aggregation
ctg_00094	*PotE*	Spermidine biosynthesis
ctg_01239	*RpoB*	beta-subunit of the RNA polymerase
ctg_00366	*PGM*	phosphoglucomutase
ctg_00120	*SpeC*	Constitutive ornithine decarboxylase
ctg_00858	*RelA*	Bi-functional protein with (p)ppGpp synthetase and hydrolase domains
ctg_00929	*BipA*	Putative GTPase
ctg_00583	*DltA*	D-alanine--poly(phosphoribitol) ligase
ctg_00264	*Mfd*	DNA repair

Evalue = 0, pident > 50%, Qcovs > 80 and genes involved in both PHI database and Virulence Factors Database are underlined.

**Table 4:** Virulence-associated genes analyzed by Virulence Factors Database.

SeqID	Accession	Gene	Name	VFcategory
ctg_00883	VFG016490	*tuf*	elongation factor Tu	Adherence
ctg_00380	VFG005582	*eno*	phosphopyruvate hydratase	Exoenzyme
ctg_00353	VFG012102	*groEL*	chaperonin GroEL	Adherence
ctg_00880	VFG048844	*gndA*	NADP-dependent phosphogluconate dehydrogenase	Immune modulation
ctg_00639	VFG043573	*dnaK*	chaperone protein DnaK	Adherence
ctg_01109	VFG000080	*clpE*	ATP-dependent protease	Stress survival
ctg_01241	VFG000079	*clpC*	endopeptidase Clp ATP-binding chain C	Stress survival
ctg_01551	VFG006720	*lap*	Listeria adhesion protein Lap	Adherence
ctg_00763	VFG047710	*carB*	carbamoyl phosphate synthase large subunit	Nutritional/Metabolic factor
ctg_00858	VFG009928	*relA*	Probable GTP pyrophosphokinase RelA	Regulation

Evalue = 0 and genes involved in both PHI database and VFDB are underlined.

**Table 5:** LPXTG-containing proteins predicted by CW-PRED database.

SeqID (Length)	Outside	Motif	Transmembrane Region	Inside
ctg_00388 (147aa)	1–109	LPQTGE	116–140	141–147
ctg_00393 (1169aa)	44–1142	LPQTGE	21–43, 1141–1162	1–20, 1163–1169
ctg_01533 (1508aa)	44–1478	LPQTGE	21–43, 1479–1501	1–20, 1502–1508

## Data Availability

*L. sae* M-11 complete genome and plasmid sequences presented in this study are openly available in the GenBank, NCBI with the accession numbers CP144759 (chromosome), CP144760, CP144761, and CP144762 (three plasmids, respectively).

## Supplementary Materials

The following supporting information can be downloaded at: https://www.mdpi.com/article/10.3390/vetsci12121204/s1, Online Resource S1: Table S1: Primers used in quantitative real-time PCR; Figure S1: Genome-atlas view of the *L. sae* M-11 chromosome and KEGG pathways; Figure S2: Western blot analysis using an anti-IBDV VP2 polyclonal antibody; Figure S3: Cell surface markers expressions of PB-MoDCs cultured for 7 d and proliferation reaction of allogenic mixed lymphocytes. Online Resource S2: Sheet S1: Summary of annotations in this study; Sheet S2: Transmembrane proteins Prediction.
