# Supplementary material for: LigiLactobacillus saerimneri M-11 as a Promising Mucosal Delivery Vector for Chickens: Genomic Insights and Discriminative Modulation of Dendritic Cell Activation
Source: Vet Sci. 2025 Dec 16;12(12):1204. doi: 10.3390/vetsci12121204 (PMC12737469; doi:10.3390/vetsci12121204)
Supplement: Supplementary file 1 [file vetsci-12-01204-s001.zip › Online Resource S1.pdf]

Online Resource 1

Table S1. Primers used in quantitative real-time PCR.

Genes	Primer sequences (5′–3′)	GenBank ID	Reference
β-actin	F:TGCTGTGTTCCCATCTATCG	NM_205518.1	
	R:TTGGTGACAATACCGTGTTCA		
MHC-Ⅱ	F:GGGGTTTACGACAGCGTCTATT	NM001001762.1	
	R:TTCCGGGTCCCACATCCT		
CD40	F:AACGCAACGCACAACACTG	EF554723.1	
	R:GTCCCTTTCACCTTCACCACA		
CD80	F:AACAGAAATGGTGGTAAGAT	NM_001079739.1	
	R:TAGAAGCAAACTGGTGGA		
CD83	F:TGTGAAGACTGGGTAATAGG	XM_418929	
	R:AGGACAACAGCAGGAAGC		
DEC-205	F:AACACGATGCCAGCTCTCAA	NM_001037836	
	R:TTGACATGAAACGTAAGCTTCCTT		[20]
IL-1β	F:GCCCTCCTCCAGCCAGAAAG	NM_204524.1	
	R:TAGCCCTTGATGCCCAGTGC		
IL-6	F:GCAGGACGAGATGTGCAA	AJ309540	
	R:CCAGGTAGGTCTGAAAGGC		
IL-12	F:TGGCCGCTGCAAACG	AY262751	
	R:ACCTCTTCAAGGGTGCACTCA		
IFN-γ	F:ATCATACTGAGCCAGATTGTTTCG	Y07922	
	R:TCTTTCACCTTCTTCACGCCAT		
TNF-α	F:GAAGCAGCGTTTGGGAGT	NM_204267.1	
	R:GTTGTGGGACAGGGTAGG		
CXCLi1	F:TGGCTCTTCTCCTGATCTCAATG	Y14971	
	R:GCACTGGCATCGGAGTTCA		

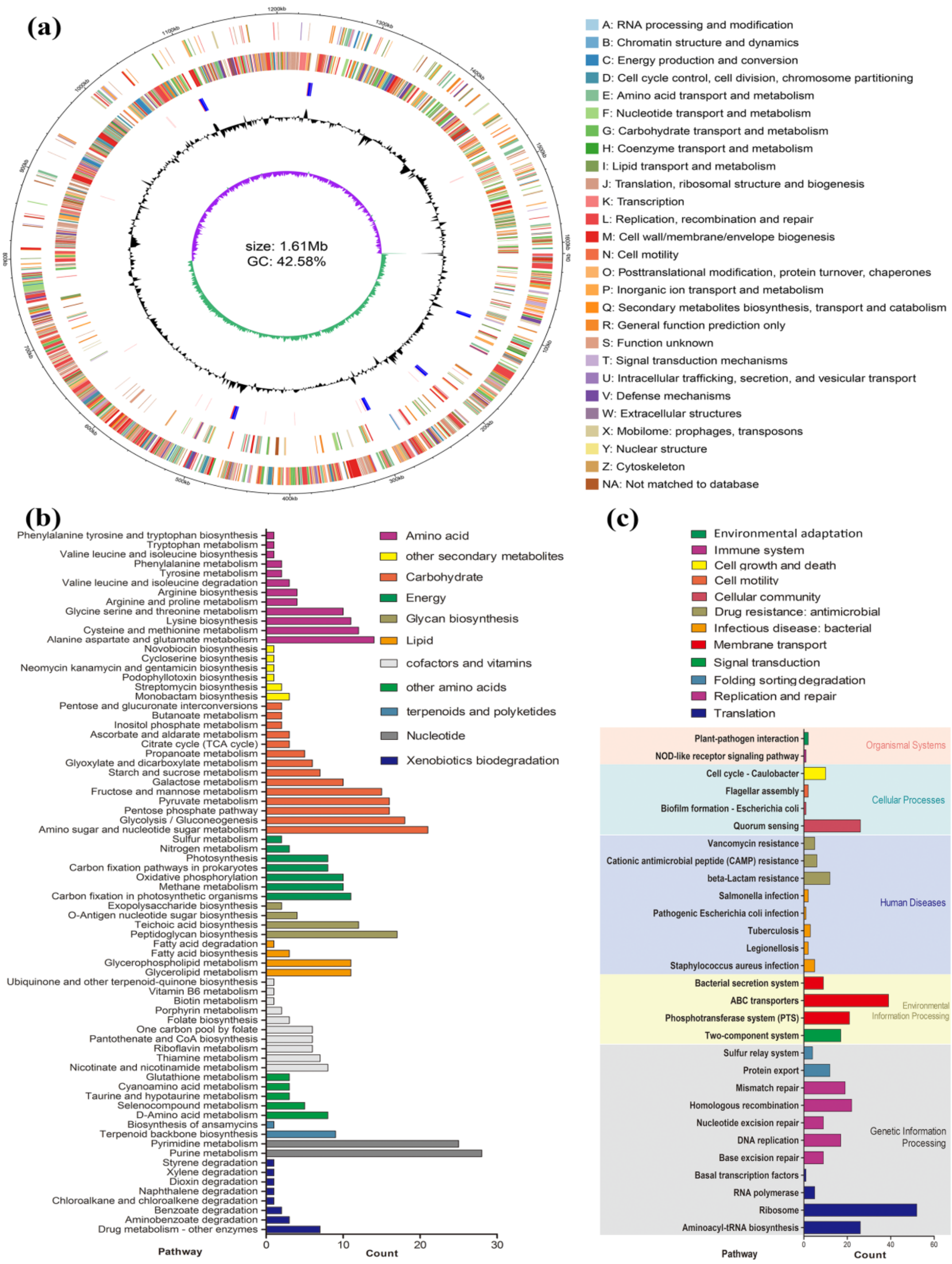

Supplementary Figure S1. Genome-atlas view of the *L. sae* M-11 chromosome and KEGG pathways. (**a**) Genome-atlas view and COG analysis. The six circles (outer to inner) are showed as follows. Circle 1 shows genome coordinate. Circles 2 and 3 are genes with different colors according to the COG classification on the forward and reverse strands. Circle 4 (purple) shows the tRNA genes (red) and the rRNA regions (blue). Circle 5 shows the GC percentage of the CDSs on the genome. Circle 6 represents the GC skew (step by 2000 bp) , G%>C% (green) and C%>G% (purple). (**b**) Pathways related to metabolism. (**c**) Pathways related to non-metabolism.

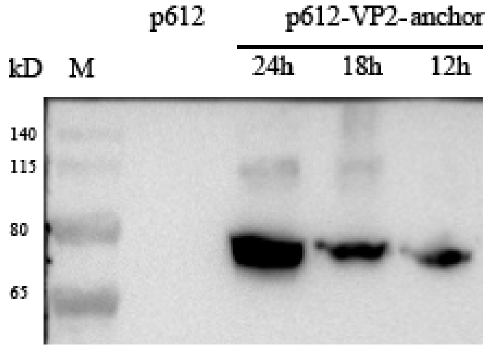

Supplementary Figure S2. Western blot analysis using an anti-IBDV VP2 polyclonal antibody.

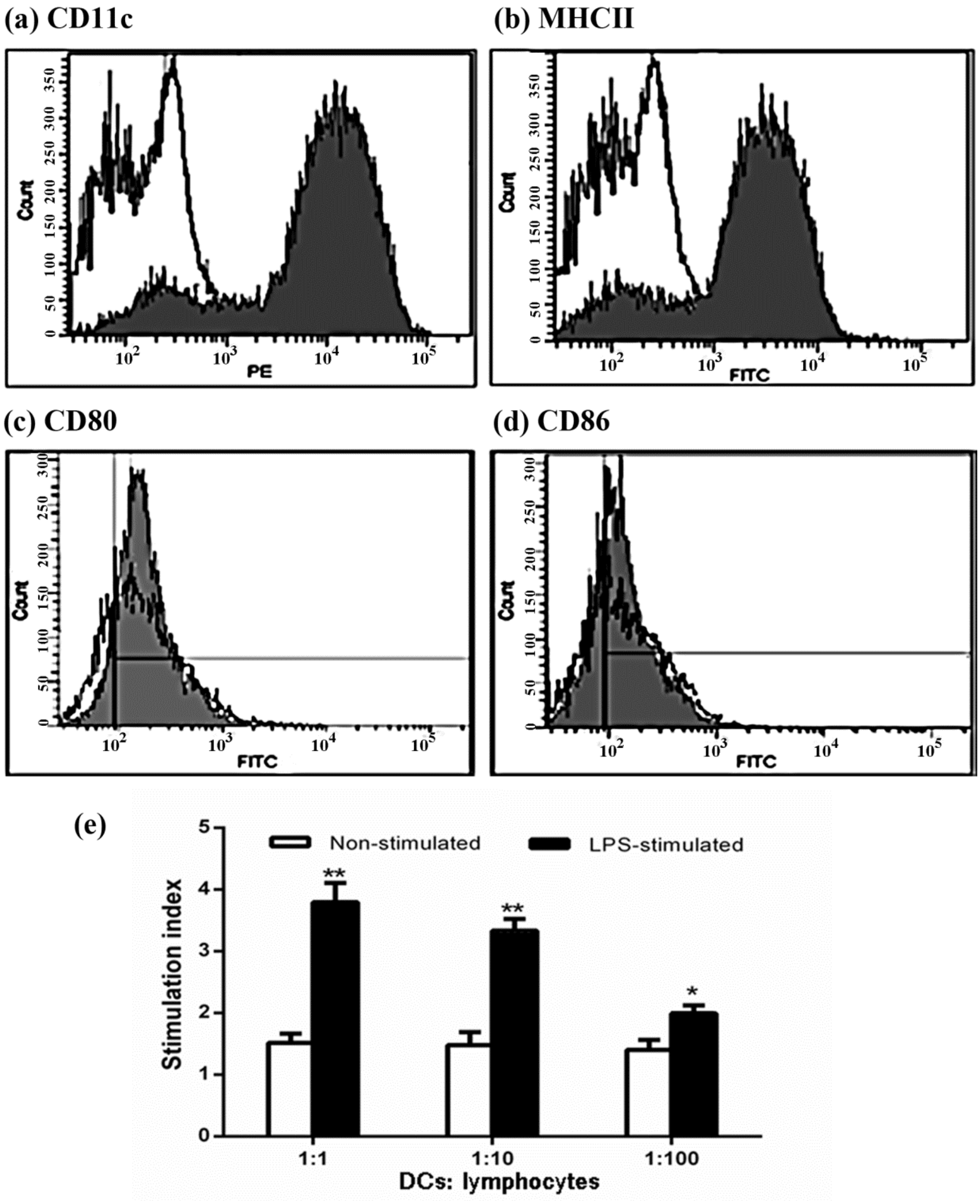

Supplementary Figure S3. Cell surface markers expressions of PB-MoDCs cultured for 7 d and proliferation reaction of allogenic mixed lymphocytes. (**a**), (**b**), (**c**) and (**d**) Cell surface markers expressions of PB-MoDCs. PB-MoDCs were stimulated with LPS (in grey) and blank medium (in transparent color with black lines); (**e**) Proliferation reaction of allogenic mixed lymphocytes.
